# Clinical Verifications

**Published:** 1891-02

**Authors:** George W. Sherbino

**Affiliations:** Abilene, Texas


### CLINICAL VERIFICATIONS.

George W. Sherbino, M. D., Abilene, Texas.

Berberis-vulgaris$^{cm}$ in Cervical Neuralgia.—A postal clerk was taken with a pain in his neck, on the right side. Pain began near the right *mastoid* process, shooting like lightning to the point of the shoulder and upper arm. *He had to keep perfectly still and extend his head to the left, putting all of the muscles on the stretch and keeping them that way.* The least motion or relaxing the muscles would cause a sudden cramp, and he would cry out.

I gave Bell. and Bry., but a close study of the case brought to light the simillimum, which was Berberis-vulg.$^{cm}$. I put one dose on the tongue, and when I called again in half an hour he could move his head in any direction, without exciting the cramping in the muscles.

LAUROCERASUS$^{1m}$ IN MASTODYNIA.—Mrs. J. was taken with a pain in the right scapula, about the centre. Constant aching (worse from motion). This pain came on after confinement in the first week. She never had it before.

Always dreads to nurse the baby, as the pain extends from the right nipple through to the sore spot in the centre of the scapula. When the baby begins to nurse she grasps the breast with the right hand, with a relaxation and contraction. A kneading motion with the hand. This she keeps up as long as the baby nurses. I asked her why she did this and she said, without this grasping her breast and punching it in that way she could not stand the pain. Laurocerasus$^{1m}$, one dose, cured.

DIOSCOREA-VIL$^{cm}$ IN UTERO-OVARIAN CARDIAC REFLEX. —I was called up at two A. M. to go and see a young lady suffering great pain. She said the pain began in the region of the womb and ovaries, then passed up to the heart, causing a constricted feeling, as if something were tight around the heart (Cact-g., Iod., Lilium-t.), causing great dyspnœa and weak, slow pulse. When the pain came on it would start from the uterus and ovaries in paroxysms, and she would scream so the neighbors could hear her. She would claw at the hypogastric region and at her heart.

I asked her where the pain seemed to go from her heart?

"*It just goes all over me.*" This led me to think of Dioscorea. I gave one dose. In five minutes she screamed no more. She was all right in the morning. I was pleased as well as the patient. Morphine was not needed, although she begged for it. I used to think this remedy had to be given in the tincture or even in the fluid extract, because some one else said so. This case shows the value of the potency.

LOBELIA$^{1m}$ IN CEPHALALGIA.—Pain commences in one temple or the other, passing around over the frontal bone to the other, or the pain may commence in both temples and seem to pass from one temple to the other. With this pain there is nausea, *dull, heavy pain, passing from one temple to the other, just above the eyebrows.* I have never known this remedy to fail with these symptoms.

SANICULA SPRING WATER$^{cmm}$ (SWAN), IN CATARRHAL OPHTHALMIA.—Last winter there came an epidemic of sore eyes in town, my two boys getting it first. One of them had so great swelling of the lids that he could with difficulty open the eyes at all, there was a constant straining or effort to keep the lid up (Caust., Gels.). I never saw so rapid and excessive secretion of pus from the eyes, it would run down upon the cheeks every few minutes, requiring to be wiped off. Great photophobia night and day. There was an amelioration in the morning, but about noon the exacerbation would come on and increase as the day advanced. In the evening he suffered so from intolerance of light he could not possibly keep his eyes open. They would now become agglutinated and remain so until next morning. Nevertheless we put sweet oil upon the eyelashes. I gave Argentum-nit. first without any benefit, but for the *cold clammy feet* I gave Sanicula.

I had a good many cases of this kind to treat, and this remedy acted splendidly.

*The cold clammy feet* (Calc-carb.), *cold clammy sweat on the back of the neck.* Both of these symptoms are in the proving and have been verified a great many times, and are never-failing indications.

SANICULA SPRING WATER$^{50m}$ (SKINNER), IN CATARRHAL OPHTHALMIA.—Mrs. P., æt. thirty-five, came to the office for medicine for sore eyes that had been sore for two or three weeks. The lids were swollen some and the eyeball red, agglutination at night, relieved by application of cold water to the eyes (Apis.). They were worse at night (Merc.). One dose of Sanicula with S. L. cured. Has had no return since.

CANTHARIS$^{39m}$ IN CHRONIC CYSTITIS.—Mr. A. J. C., æt. seventy-two, came to consult me for bladder trouble, which he has had for a number of years. Has been taking great deal of stuff from the regular profession, but constantly growing worse.

*Symptomatology.*—Urging desire to urinate, passing only a few drops at a time of highly-colored urine mixed with blood. Urine very scanty with a cloudy sediment, and at times there is a white sediment looking like fragments of mortar. There was urging to urinate from the least quantity of urine in the bladder. It was worse from walking and better from sitting or lying down.

Painfulness from riding in a wagon or on horseback. Sometimes the urine suddenly stops, " like stone in the bladder."

He got one dose of Canth$^{39m}$ dry. In a week he wrote me that he was very much better. I sent him two prescriptions S. L. by mail afterward and he remains well now for six months. I gave an unfavorable prognosis from the symptoms, as I thought they all pointed to stone in the bladder and taking into consideration his old age.

